# Radiation-Induced Bystander Effect and Cytoplasmic Irradiation Studies with Microbeams

**DOI:** 10.3390/biology11070945

**Published:** 2022-06-21

**Authors:** Ziqi Zhang, Kui Li, Mei Hong

**Affiliations:** 1 College of Life Sciences, South China Agricultural University, Guangzhou 510642, China; zhangziqi19@stu.scau.edu.cn (Z.Z.); lk0317@stu.scau.edu.cn (K.L.); 2 Guangdong Provincial Key Laboratory of Protein Function and Regulation in Agricultural Organisms, South China Agricultural University, Guangzhou 510642, China

**Keywords:** ionizing radiation, microbeams, radiation-induced bystander effect, cytoplasmic irradiation

## Abstract

**Simple Summary:**

Microbeams are useful tools in studies on non-target effects, such as the radiation-induced bystander effect, and responses related to cytoplasmic irradiation. A micrometer or even sub-micrometer-level beam size enables the precise delivery of radiation energy to a specific target. Here we summarize the observations of the bystander effect and the cytoplasmic irradiation-related effect using different kinds of microbeam irradiators as well as discuss the cellular and molecular mechanisms that are involved in these responses. Non-target effects may increase the detrimental effect caused by radiation, so a more comprehensive knowledge of the process will enable better evaluation of the damage resulting from irradiation.

**Abstract:**

Although direct damage to nuclear DNA is considered as the major contributing event that leads to radiation-induced effects, accumulating evidence in the past two decades has shown that non-target events, in which cells are not directly irradiated but receive signals from the irradiated cells, or cells irradiated at extranuclear targets, may also contribute to the biological consequences of exposure to ionizing radiation. With a beam diameter at the micrometer or sub-micrometer level, microbeams can precisely deliver radiation, without damaging the surrounding area, or deposit the radiation energy at specific sub-cellular locations within a cell. Such unique features cannot be achieved by other kinds of radiation settings, hence making a microbeam irradiator useful in studies of a radiation-induced bystander effect (RIBE) and cytoplasmic irradiation. Here, studies on RIBE and different responses to cytoplasmic irradiation using microbeams are summarized. Possible mechanisms related to the bystander effect, which include gap-junction intercellular communications and soluble signal molecules as well as factors involved in cytoplasmic irradiation-induced events, are also discussed.

## 1. Introduction

It has been generally accepted by radiation-biology studies that the effects of radiation are mediated by direct damage to nuclear DNA [1]. A major paradigm shift in radiation biology in the past two decades has resulted from the studies of a radiation-induced bystander effect (RIBE), in which cells that are not themselves directly in the path of ionizing radiation also exhibit biological responses, likely by receiving signals transmitted from the hit cells [2]. A bystander effect is considered as an important non-target impact and usually results in deleterious responses. The capability of microbeams that can precisely deliver radiation, without damaging the surrounding area, provides a useful tool for exploring the underlying mechanisms of such kind of effects. Irradiation of the extranuclear target, which is termed cytoplasmic irradiation, may also be considered as a kind of non-target effect. For decades, biologists and geneticists have been interested in the differential biological effects resulting from nuclear versus cytoplasmic irradiation. The availability of microbeams that can deposit energy precisely to a part of the cell (such as the cytoplasm or nucleus) makes it possible to investigate the damage caused by irradiation of the nuclei versus the cytoplasm, in a separate manner [3].

## 2. Non-Target Effects

### 2.1. Radiation-Induced Bystander Effect

The radiation-induced bystander effect (RIBE) was first reported in 1992, with Chinese hamster ovary cells irradiated with an extremely low dose of alpha particles. It was found that when less than 1% of the cell nuclei were traversed by an alpha particle, 30% of the cells showed an increased frequency of sister-chromatid exchanges [4]. For more than two decades, accumulating evidence has shown that these effects are the results of a dynamic and integrated process that is initiated by irradiated cells and transmitted to the neighboring cells [5]. In vitro and in vivo studies on RIBE have been carried out with high and low LET radiation [6,7,8], normal and tumor cells [9,10], and regular two-dimensional (2D) monolayer cell systems and three-dimensional (3D) tissue systems [11]. Changes observed in the bystander cells included reduced clonogenic survival, increased sister-chromatid exchange, micronuclei formation, apoptosis, and alteration of gene expression and RNA-transcript level. It was proposed that irradiated cells induced RIBE by transmitting molecular signals through direct contact, such as intercellular gap junctions, or by releasing diffusible factors such as cytokines and chemokines [1]. Moreover, it was demonstrated that bystander cells also generated signals and affected the responses of irradiated cells, so the existence of a reciprocal dialogue between these two populations of cells was proposed [12]. In vitro systems for the studies of RIBE mainly include low doses of irradiation of monolayer cell cultures, medium-transfer experiments in which the culture medium of irradiated cells is harvested and used to treat unexposed cells, irradiation of partial-shielded monolayer cultures, co-culture irradiation systems using a transwell chamber with a porous membrane, and microbeams that directly irradiate precise locations in the cell cultures [13]. A bystander effect is usually considered as a local communicative effect at the primary site over a few millimeters or cellular diameters. While, systematically, a similar effect transmitted for a long-distance (up to tens of centimeters outside of the irradiated field) is termed an abscopal effect, which is mediated through immunogenic responses and is an important factor that need to be considered in exploring the strategy of collaborating ionizing radiation with cancer immunotherapy in oncology practice [5]. 

### 2.2. Cytoplasmic Irradiation

It has been generally accepted in radiation biology that genotoxic effects, such as mutations and carcinogenesis, which are attributed to ionizing radiation exposure, mainly result from direct damage to the nuclei. Therefore, the consequence of cytoplasmic irradiation is mainly underestimated. For example, though the likelihood of the bronchial or lung cells to get an alpha-particle traversal through the cytoplasm is much higher than the nuclei in the environmental radon exposure, the contribution from the cytoplasmic traversal is usually ignored in the current radiation-risk-estimation model [3]. Irradiation of an extranuclear target, i.e., the cytoplasm, has been demonstrated to result in mutations in the nuclei. It was shown that cytoplasmic irradiation of human–hamster-hybrid (A_L_) cells with eight alpha particles led to around a threefold increase in CD59^−^ mutations, while inflicting minimal cytotoxicity [14]. More recent studies demonstrated that cytoplasmic irradiation is associated with mitochondrial dysfunction [15] and autophagy [16]. Cytoplasmic irradiation can also induce a bystander effect, exhibiting genotoxic effects such as mutation in both directly and indirectly irradiated cells. Reactive oxygen species/reactive nitrogen species (ROS/RNS) are demonstrated to play an important role in the process, and multiple signaling pathways are involved [3]. 

## 3. Current Development of Microbeams

The first microbeam apparatus was developed in 1912, and the first experiment carried out with a microbeam setting was to study the effect of proton exposure on the cell-division process in 1953 [17]. A microbeam is a beam of radiation with a diameter at the micrometer or sub-micrometer level so that the energy can be precisely deposited at specific locations within a biological target. Due to its small beam size, microbeam irradiators became a useful instrument, solving problems such as irradiation imprecision, low-dose radiation, and accuracy challenges [17]. The two most popular approaches to maintain the small size of microbeams are collimation and magnetic focusing. The magnetic-focusing systems are more preferable in heavy-ion irradiators because they do not have the scattering problems of collimators. Magnetic or electrostatic focusing devices can narrow down charged-particle beams to a few tens of nanometers under vacuum conditions. Additionally, the field strength required for focusing is independent of the particle mass and charge of the accelerator, especially in the electronically focused system [18]. 

Charged-particle microbeams (CPMs) have been developed worldwide for the targeted micro-irradiation of living cells, which provide unique features for radiation biology studies. Major microbeam facilities nowadays include the Radiological Research Accelerator Facility (RARAF) of Columbia University (particles: proton (p) and alpha (α), energy range: 1–5 MeV, beam size: 0.8 μm) [19]; the Ion Beam Center at the University of Surrey (particles: p to calcium, energy range: 0.5–12 MeV, beam size: 1 μm) [20]; the heavy-ion microbeam systems at JAERI, Takasaki, Japan (particles: α, C, Ne, Ar, energy range: 11.5–18.3 MeV/u, beam size: 5 μm) [21]; the GSI heavy ion microbeam facility at Darmstadt, Germany (particles: C to U, energy range: 1.4–11.4 MeV/u, beam size: ≤2 μm) [22]; SNAKE at Munich, Germany (particles: mainly C and O, also p, α, Li, Si, Cl, and I, energy range: 0.5–100 MeV/u, beam size: 0.7 μm in x- and 0.8 μm in y-direction) [23]; and the Heavy Ion Research Facility (HIRFL) operated by the Institute of Modern Physics of the Chinese Academy of Sciences in Lanzhou, China (particles: C, energy range: several MeV/u to 100 MeV/u, beam size: 3 μm × 5 μm) [24]. Using CPM, a precise number of ions can be delivered to individual targets with a micrometer lateral resolution. Hence, it is easy to focus on a very small target and deliver radiation without damaging the surrounding area. The radiation can be applied to target sub-cellular compartments with micrometer precision. These unique features cannot be achieved by other irradiators [25], making microbeams a useful radiation source in studies of the bystander effect and cytoplasmic irradiation. 

## 4. Studies of Non-Target Effects with Microbeams

### 4.1. Bystander Effect Observed with Microbeam Experiments

Although shielding or medium-transfer systems could be utilized for radiation-induced bystander studies, microbeams provide precise targeting and dose delivery to individual cells, which makes them particularly suitable for the investigation of the bystander effect [2]. The first study of RIBE using a microbeam irradiator was performed more than two decades ago with primary human fibroblast [26]. It was demonstrated that when only four cells were traversed by alpha particles, the micronucleated and apoptic cells generated in the cell population were much higher than expected from only a direct effect, suggesting the presence of RIBE in the non-irradiated cells. A radiation-induced bystander effect was also shown in different biological systems with different radiation types and microbeam settings in subsequent studies. For example, by using the human–hamster-hybrid A_L_ cells, Zhou and co-workers demonstrated that when 20% of cells were randomly selected and irradiated with 20 alpha particles, mutation on the CD59 locus was three-fold higher than expected [27]. Using the oncogenic transformation ratio as an end point, Sawant and co-workers also showed that when 10% of the pluripotent stem cells C3H10T1/2 were irradiated through the nuclei with an alpha particle, the resulting transformation frequency was comparable to that observed when every cell on the dish was exposed to the same number of alpha particles [28]. By using a HeLa-FUCCI (Fluorescent Ubiquitination-based Cell Cycle Indicator) system, Kaminaga and co-workers investigated how bystander cells responded to soft X-ray-microbeam irradiation. Effects such as cell-cycle retardation, explosive cell death, or cell fusion were observed in 15% and 26% of the bystander cells for 10 and 20 Gy irradiation, respectively. Moreover, these serious effects appeared in daughter or granddaughter cells from a single-parent cell as well [29]. When human keratinocyte cells with HPV-G were directly irradiated with a charged particle microbeam, apoptosis was induced and the mitochondrial-membrane potential was reduced in the bystander cells. Additionally, an increased ROS level was observed, followed by increased expression of the anti-apoptotic protein Bcl-2 and release of cytochrome c. The study also compared the bystander effect induced by microbeam irradiation to that of the medium-transfer method and concluded that similar signaling pathways are involved [30]. Although most RIBE studies have been focused on the radiobiological changes observed in bystander cells in response to the signals excreted from irradiated cells, recent studies have also utilized microbeams to investigate the bidirectional and criss-cross bystander communications between cancer cells and normal cells. For instance, when γ-H2AX foci fluorescence, a measure of DNA double-strand breaks (DSBs), was utilized as the observation endpoint, it was found that in the co-culture of lung-cancer cells A549-A549, the proton-irradiated A549 cells could exert a damaging effect on the bystander cancer cells. On the other hand, when A549 cells were co-cultured with WI38, a human-diploid fibroblast cell line, irradiated A549 exhibited no effect on the bystander WI38 cells. The fibroblast cells may have induced inverse protective signaling in the irradiated A549 cells instead. Interestingly, the irradiation of WI38 did not trigger any bystander effect on either A549 or WI38. This research on the reciprocal signaling of bystander cells may be relevant to the therapeutic outcome of cancer radiotherapy [31]. 

Besides different cell lines, RIBE observed with microbeam settings has also been reported in three-dimensional tissues. In a study using a model of the human epidermis that consists of multilayer epidermal keratinocytes, Belyakov and co-workers described bystander responses in a 3D, normal human-tissue system. A charged-particle microbeam delivered defined numbers of alpha particles to specific locations, and unirradiated cells up to 1 mm distant from irradiated cells exhibited a 1.7-fold increase in micronuclei formation as well as a 2.8-fold enhancement of apoptosis compared to the control These findings suggest that bystander signals may be transferred over a long distance in human tissue [32]. Studies of human tissue with charged-particle microbeams also found that DNA double-strand breaks are involved in bystander responses and that DSBs preceded downstream effects, such as micronucleus formation and apoptosis. Moreover, post-irradiation genomic instability was observed in bystander cells, suggesting that they are more prone to become cancerous compared to those unaffected cells [33].

Quite a few studies also have used the *Caenorhabditis elegans* system to study the bystander effect with microbeam irradiators. By using a collimated microbeam of 220 MeV ^12^C^5+^ particles delivered from the azimuthally varying field (AVF) cyclotron, it was found that the cell-cycle arrest and apoptosis induced by DNA damage were only observed in locally irradiated regions of the nematode, while little of the bystander effect was observed [34]. However, a later report using a nematode *C. elegans* strain with a Green Fluorescent Protein (GFP) reporter for the hsp-4 heat-shock gene demonstrated that when 3 MeV protons were precisely delivered to a site in the tail of the nematode, enhanced expression of the GFP reporter was observed in a proton number-dependent manner after 24 h, at distances more than 100 μm from the irradiated site, suggesting the presence of RIBE [35]. Studies with a proton microbeam also demonstrated that irradiation of the posterior pharynx bulbs and tails of *C. elegans* enhanced the level of germ-cell apoptosis in bystander gonads. Moreover, the level of DNA damage was increased in bystander germ cells and genomic instability was observed in the F1 progeny [36]. 

The first study on RIBE induced by microbeam irradiation in an intact living mammal was reported in 2015 [37]. The ear of a C57BL/6J mouse was irradiated along a 35 μm wide and 6 mm long line. It was found that γ-H2AX foci were formed in one of the two epidermal layers of the mouse ear after irradiation, and a higher number of cells exhibited foci than from only the direct irradiation. In addition, the average width spanned by γ-H2AX-positive cells exceeded 150 μm, though the proton-irradiated path was only 35 μm wide. Abscopal effect, which can be regarded as a bystander effect in the whole organism level, was reported with animal models as well. When the up-regulation of apolipoprotein E (ApoE) and L-plastin (lymphocyte cytosolic protein 1) served as endpoints, and a collimated microbeam of carbon ions (250 µm diameter) was used as the irradiation source, irradiation of the embryonic midbrain of medaka fish (*Oryzias latipes*) resulted in the up-regulation of L-plastin in the microglia located in the irradiated area. However, the activated microglia may move from the irradiated area and spread to the embryonic brain, causing an abscopal effect following irradiation [38]. The immune system is considered as a key machinery for the abscopal effect. It was demonstrated that signals leading to abscopal and bystander effects were observed in immunocompromised mice exposed to pencilbeam and microbeam synchrotron radiation. The authors proposed that innate immune response alone may be enough to trigger the out-of-field effects [39]. 

### 4.2. Cytoplasmic Irradiation with Microbeams

Microbeams can precisely deposit energy to specific parts of the cell, such as the cytoplasm or nucleus, making it possible to differentiate the biological effects from damage to different parts of the cell [3]. As early as 1964, Dendy and Smith reported that irradiation of the cytoplasm with ultraviolet or alpha-particle microbeams resulted in a progressive reduction in the DNA-synthesis rate, though to a lesser extent than that of nuclear irradiation [40]. Most of the effects induced by cytoplasmic irradiation in early reports were relatively minor compared to those induced by nuclear irradiation, hence, the idea that the genotoxic effects of ionizing radiation are due mainly to direct damage to the nucleus still prevailed. In 1999, Wu and co-workers demonstrated that the irradiation of cellular cytoplasm of A_L_ cells with alpha particles resulted in CD59^−^ mutations in the nucleus. Cytoplasmic irradiation resulted in less cell toxicity, as more than 70% of cells survived even after their cytoplasm was irradiated with 32 alpha particles. The survival fractions for nuclear irradiation with the same number of alpha particles, however, was merely 0.1%. The principal classes of mutations induced by cytoplasmic irradiation are mostly point mutations, similar to those of spontaneous origin, but entirely different from those of nuclear irradiation. Therefore, cytoplasmic irradiation may pose a higher risk than nuclear traversal, since the mutagenicity generated is accomplished by far less killing of the target cells [14]. DNA double-strand breaks were found to be induced by cytoplasmic irradiation as well. For example, Konishi and co-workers compared the radiobiological effects between nuclear and cytoplasmic irradiation with a microbeam that can target different cellular parts with the desired number of protons. It was found that in the cytoplasmic-irradiated cells, though the γ-H2AX level was comparable to that of the non-irradiated control at 1 h after irradiation, the DSB marker increased significantly at a later time point (4-h post-irradiation). Moreover, elevation of the γ-H2AX foci was proportional to the number of protons delivered [41]. Cytoplasm irradiation may also cause dysfunction of the mitochondria. When the cytoplasm of human small-airway epithelial cells was targeted using an alpha particle microbeam irradiator with a beam width of 1 μm, mitochondrial fragmentation and decreased cytochrome c oxidase and succinate dehydrogenase activity were observed, indicating a reduction in respiratory-chain function [15]. Furthermore, the mitochondrial dysfunction induced by cytoplasmic irradiation resulted in autophagy activation, which degraded dysfunctional mitochondria to maintain cellular-energy homeostasis. An interesting finding of the study was that the activation of autophagy was only detected in cytoplasmic irradiated but not in nuclear irradiated cells, suggesting a protection attempt of cytoplasmic irradiated cells from toxic effects [16]. Changes in mitochondrial area was also demonstrated with cytoplasmic irradiation. Kaminaga et al. irradiated normal human diploid cells with a synchrotron X-ray microbeam and found that cytoplasmic irradiation temporarily enlarges the mitochondrial area, with high membrane potential. However, no significant change was observed in the total mitochondrial area. On the other hand, cell-nucleus irradiation promoted an increase both in the mitochondrial areas with high membrane potential and low membrane potential, which resulted in an apparent increase in the total mitochondrial area [42]. Targeted cytoplasmic irradiation was found to induce a metabolic shift in human small-airway epithelial cells (SAE) as well. Twenty-four hours after cytoplasmic irradiation with an alpha particle microbeam, an increased mRNA expression level of the key glycolytic enzymes as well as lactate secretion was observed in SAE cells, suggesting a shift from an oxidative to a glycolytic phenotype [43]. 

Cytoplasmic irradiation also led to a bystander effect. Shao and co-workers showed that when the cytoplasm of one cell within a radioresistant glioma population was precisely transversed by a single alpha particle, the micronuclei yield was increased by 36% and 78% for the neighboring, non-irradiated glioma and the fibroblast bystander cells, respectively. Interestingly, the yield of the bystander-induced micronuclei was comparable between cytoplasmic and nuclear irradiation [44]. Targeting only the cytoplasm of HeLa cells with alpha particles also elicits the formation of p53 binding protein 1 (53BP1) foci at DSB sites in both irradiated and bystander cells. In addition, the foci formed were independent of the dose or the number of cells irradiated. Therefore, the underlying mechanisms of the bystander effect induced by cytoplasmic irradiation may be similar to that elicited by nuclear targeting, since the levels of response were not significantly different from each other [45]. A cytoplasmic-irradiation-induced bystander effect may also lead to genomic instability. When persistent chromosomal changes were analyzed in the progeny of irradiated and bystander A_L_ cells with multicolor banding (mBAND), after the nucleus or cytoplasm was precisely hit by alpha particles, it was demonstrated that the progeny of both nuclear- and cytoplasmic-targeted cells exhibited a significantly higher number of metaphase changes in human chromosome 11 than the controls. Similar chromosomal changes were detected among the progeny of the bystander cells as well. These results suggest that the genomic instability induced after irradiation may be decided mainly by initial radiation damage events and may not necessarily require direct damage to the cell nucleus [6]. 

### 4.3. Mechanistic Studies of Non-Target Effect with Microbeams

#### 4.3.1. Mechanisms of Bystander Effect Induced by Microbeam Irradiation

It has been shown that gap-junction intercellular communications (GJIC) and signal molecules released by irradiated cells are two essential factors involved in RIBE. Gap-junction communication is believed to play major roles in confluent cultures, where irradiated and non-irradiated cells are physically in contact with each other; while in sparsely populated cultures, signal molecules are excreted into the medium from irradiated cells, causing bystander responses [46]. 

Studies with microbeams demonstrated that gap-junction communications play important roles in RIBE between cancer and normal cells. When confluent cells were exposed to different kinds of microbeams, including X-ray, carbon ion, neon ion, or argon ion, it was found that a greater fraction of cells formed micronuclei than expected, based on the fraction of cells targeted by primary radiation, suggesting the presence of a bystander effect. When 18-α-glycyrrhetinic acid (AGA), an inhibitor of GJIC, was applied in the cell culture, the induction of micronuclei was suppressed at high mean absorbed doses of heavy-ion irradiation, but no significant difference was observed in the X-ray irradiation, which implicated GJIC as involved only in the bystander effect induced by high-LET irradiation. A bystander effect derived from low-LET irradiation, on the other hand, may be meditated by secreted factors from the irradiation cells, since non-irradiated cells exposed to the growth medium harvested from X-ray-irradiated cells exhibited the bystander effect in the form of excess micronucleus formation [47]. When proton microbeams were applied to confluent A549 cells, toxicity effects such as colony and micronucleus formation as well as chromosome aberration in bystander A549 cells were suppressed, when the gap junction was inhibited, suggesting involvement of GJIC [48]. Gap-junction communication may also play a role in a secondary bystander effect. A radiation-induced secondary bystander effect is induced by the primary bystander cells that produce secondary bystander signals to their neighboring cells, resulting in altered biological endpoints. After the primary bystander cells were generated with human lung-cancer cells A549, using a single dose or fractionated doses of a proton, the bystander cancer cells were seeded on the top of the insert with secondary bystander WI-38 cells. It was found that fractionated doses caused 20% less micronucleus formation in the secondary bystander WI-38 cells than a single radiation dose. However, the presence of AGA in the co-culture system could eliminate the secondary bystander effect [49]. 

Quite a few studies with microbeams demonstrated that, in addition to GJIC, other factors are involved in RIBE. In a study in which a precise number of helium-3 (^3^He^2+^) particles were delivered with a microbeam irradiator through the center of the nucleus or the cytoplasm, rapid calcium fluxes were observed in the non-irradiated T98G glioma cells and AG01522 fibroblasts that were transferred to the conditioned medium of the irradiated cells or co-cultured with the irradiated cells. Micronuclei were induced in bystander cells, but the effect was dramatically suppressed with the treatment of calcicludine (CaC), a calcium-channel blocker. Further, the nitric-oxide-synthase (NOS) inhibitor aminoguanidine and the reactive oxygen species (ROS)-blocker dimethyl sulfoxide (DMSO) inhibited calcium fluxes and the bystander effect in T98G and AG01522 cells, respectively, suggesting the involvement of these bioactive molecules in RIBE [50]. Studies have also demonstrated that mitochondria serve important roles in the bystander effect. After generating mitochondrial DNA-depleted ρ^0^ cells by treating parental human skin fibroblasts (ρ^+^) with ethidium bromide, it was found that ρ^0^ cells exhibited a higher mutagenic response than ρ^+^ cells, if a fraction of the same population in confluent monolayers was irradiated with lethal doses. However, if the ρ^0^ and ρ^+^ populations were mixed and only one population of cells were irradiated with a lethal dose delivered by an alpha-particle microbeam, a decreased bystander mutagenesis was observed in bystander cells, suggesting communication signals between these two types of cells with different mitochondria content. Nuclear factor-*κ*B (NF-*κ*B) inhibitor Bay 11-7082 and nitric oxide scavenger 2-(4-carboxyphenyl)-4,4,5,5-tetramethylimidazoline -1-oxyl-3- oxide significantly decreased the mutation frequency in both types of bystander cells. Additionally, NF-*κ*B activity as well as level of cyclooxygenase-2 (COX-2) and inducible NO synthase (iNOS) were lower in bystander ρ^0^ cells, suggesting that the mitochondria-dependent NF-κB/iNOS/ NO and NF-κB/COX-2/prostaglandin E2 signaling pathways are essential for RIBE [51]. In another study using Ar-ion microbeams, NO was identified as an important molecule mediating the signals in RIBE. Activation of NF-κB and Akt was observed in both irradiated and bystander cells, but the induction depended on the post-irradiation incubation time. COX-2 level was elevated in bystander cells but not in directly irradiated cells [52]. A more recent study differentiating bystander effects induced by nuclear or cytoplasm irradiation revealed that subcellular organelles, such as mitochondria and endoplasmic reticulum (ER), play different roles in RIBE with respect to nuclear and cytoplasmic irradiation. Treatment with both the mitochondria-activation-inhibitor rotenone and the siRNA of ER-regulation-protein BiP lead to the reduced bystander response to the nuclear irradiation, but an enhanced bystander effect was induced by cytoplasmic irradiation [53]. 

**Figure 1:**
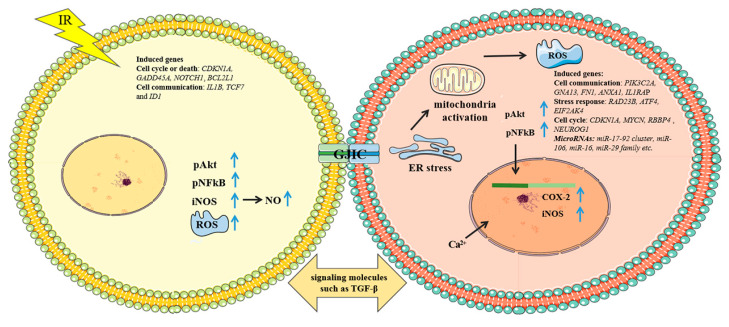
Regulatory factors involved in bystander effects induced by microbeam irradiation. Please refer to main text for detailed information.

Altered gene-expression levels were observed in RIBE induced by microbeams. Change in cyclin-dependent kinase inhibitor 1A (*CDKN1A*) mRNA was shown both in directly irradiated and bystander fibroblasts, following the microbeam delivery of 0 or 10 α-particles per cell to 50% of the cells in a population [54]. A microarray analysis of irradiated and bystander fibroblasts in confluent cultures with carbon-ion microbeams demonstrated that 874 probes exhibited more than 1.5-fold changes in bystander cells. Among them were genes related to cell communication (*PIK3C2A, GNA13, FN1, ANXA1*, and *IL1RAP*), stress response (*RAD23B, ATF4*, and *EIF2AK4*) and the cell cycle (*MYCN, RBBP4*, and *NEUROG1*). On the other hand, genes related to the cell cycle or death (*CDKN1A, GADD45A, NOTCH1*, and *BCL2L1*) and cell communication (*IL1B, TCF7*, and *ID1*) were induced in irradiated cells but not in bystander cells. These results suggested that the signals communicating between irradiated and bystander cells led to intracellular signaling and a transcriptional stress response with the bystander cells [55]. MicroRNAs are likely to be involved in mediating RIBE as well. A microRNAome analysis of the bystander tissues was carried out in a 3D artificial-human-tissue system with an α-particle microbeam irradiator. The study found that change of miRNAs in bystander tissues may lead to the alteration of biological end points, such as apoptosis, cell-cycle deregulation, and DNA hypomethylation. A significant up-regulation of c-MYC and the miR-17-92 cluster was shown, along with the down-regulation of transcription factor E2F1 in bystander tissues. In addition, the up-regulation of miR-106 was correlated with the down-regulation of retinoblastoma protein RB1, suggesting the switching of bystander tissues to a proliferative state. On the other hand, the level of the anti-apoptotic protein Bcl-2 in bystander tissues was reduced due to the up-regulation of miR-16, causing a dysregulation of cell death. An increased level of miR-29 family members was observed along with the down-regulation of DNA methyltransferase 3 alpha (DNMT3a) and myeloid-cell leukemia 1 (MCL1), which in turn affected DNA methylation and apoptosis [56]. The above-mentioned RIBE mechanisms are summarized in Figure 1. 

#### 4.3.2. Mechanisms of Effects Derived from Cytoplasmic Irradiation

It was found that ROS and RNS play essential roles in the effects caused by cytoplasmic irradiation. Using an alpha-particle microbeam, Hong and co-workers showed that the cytoplasmic irradiation of A_L_ cells induced the formation of 8-hydroxy-2’-deoxyguanosine (8-OHdG), an indicator of DNA oxidative damage. Additionally, application of nitric-oxide-synthase inhibitor N^G^-methyl- L-arginine (L-NMMA) suppressed the mutant fraction. As downstream products such as 4-hydroxynonenal were generated from free radicals and oxidants, an increased level of COX-2 and the elevated activity of extracellular signal-related kinase (ERK) were detected [57]. Nitric oxide may be involved in cytoplasmic irradiation and induced a bystander effect as well. Elevated NO level was detected in glioma cells where the cytoplasma was hit by an alpha particle. The response in the neighboring non-irradiated glioma and fibroblast bystander cells, however, was entirely inhibited when NO scavenger 2-(4-carboxyphenyl)-4,4,5,5-tetramethyl-imidazoline-1-oxyl-3-oxide or the membrane-disruptive agent filipin was applied, implicating that cytoplasmic traversal induced a bystander effect that is mediated through glycosphingolipid-enriched membrane microdomains [44]. Further study with a similar setting demonstrated that transforming-growth factor β1 (TGF-β1) is an important downstream-signaling molecule of radiation-induced NO and is excreted from the irradiated cells to induce RIBE [58].

**Figure 2:**
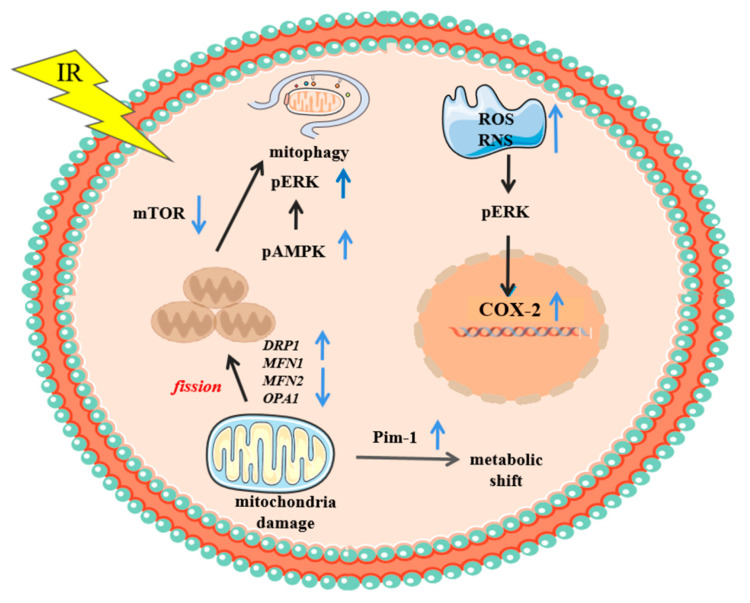
Regulatory factors involved in cytoplasmic-irradiation-related effects resulted from microbeam irradiation. Please refer to main text for detailed information.

Current studies also demonstrated that mitochondria and autophagy are involved in cytoplasmic-irradiation effects. As mentioned in the previous section, the cytoplasmic irradiation of human small-airway epithelial cells with alpha particles led to mitochondrial fragmentation. Mitochondrial-fission gene *DRP1* was up-regulated, while the expression of fusion genes *MFN1*, *MFN2*, and *OPA1* was reduced, implicating abnormal mitochondrial dynamics after cytoplasmic irradiation. When cells were treated with mitochondrial-division-inhibitor mdivi-1, DRP1 was suppressed and mitochondrial fission was reduced. Moreover, irradiated cells also exhibited an increased ROS level that could be quenched by the radical scavenger DMSO [15]. Further study also revealed that the mitochondrial dysfunction induced by cytoplasmic-irradiation-activated autophagy, which degraded the damaged mitochondria to maintain cellular-energy homeostasis. Cytoplasmic-irradiation-induced autophagy was oxyradical dependent, and DRP1 is involved in the process. Further, mitochondrial fission led to the phosphorylation of the AMP-activated protein kinase (AMPK) and its downstream target ERK1/2, which may act as a sensor for mitochondrial stress. On the other hand, the mammalian target of rapamycin (mTOR) signaling, which acts as an inhibitor of autophagy initiation, was found to be suppressed [16]. An RNA-sequencing analysis compared genes that were responsive to cytoplasmic versus nuclear irradiation and found that the expression level of Pim-1, a proto-oncogene kinase that is related to glycolysis, was significantly increased only in cytoplasmic-irradiated cells. Mdivi-1 greatly suppressed the induction of Pim-1, suggesting up-regulation of the kinase as a downstream effect of mitochondrial dysfunction. The selective Pim-1 inhibitor Smi-4a significantly suppressed lactate production and glucose uptake in cytoplasmic-irradiated cells, suggesting the involvement of Pim-1 in the metabolic shift induced by cytoplasmic irradiation [44]. The above-mentioned molecular and cellular mechanisms related to cytoplasmic irradiation are summarized in Figure 2. 

## 5. Perspectives

The capability to deliver an exact number of particles to a precise target has made microbeam irradiators a precious tool in studies related to non-target effects, especially for cytoplasmic-irradiation-induced effects. Information from the above-mentioned studies have unambiguously demonstrated that the radiation energy deposited on extranuclear targets also results in detrimental consequences for the cell. Since cytoplasm occupies a major portion of the cell, the related effect cannot be ignored when studying radiation damage. Further development of the microbeam techniques will enable acquisition of more in-depth knowledge of cytoplasmic-irradiation-derived effects and a better evaluation of radiation risk. Studies have shown that irradiated and bystander cells cross-talked with each other and that bystander cells not only accept signals from irradiated cells, they secreted responding factors that may affect irradiated cells as well. Information on the underlying mechanisms, however, is quite limited and further investigation is warranted. Little published research has utilized three-dimensional systems or live animals, especially mammals, to study RIBE with microbeams. With the development of spheroids and a better imaging system for microbeam facilities, a more comprehensive and thorough understanding of the radiation-induced bystander response in 3D systems with multicellular structure will be attained. This information will be beneficial to clinical practice for clarifying observations related to the abscopal effect, which may contribute to normal tissue injury and tumor recurrence. 

Microbeams are also superior in studies of low-dose/low-dose-rate irradiation, which is often the case in space flights. High-LET irradiation, especially related to heavy ions, is unavoidable in a space environment and is often a risk of concern during long-term space flights. The existence of a non-target effect could significantly increase the detrimental effect of radiation that may be encountered by astronauts. However, only a few reports have focused on the investigation of the non-target effect utilizing heavy-ion microbeams. The development of more advanced microbeam facilities will undoubtedly provide valuable information about the non-target effects caused by simulated space radiation, and a more detailed understanding of the corresponding mechanism(s) will help us to better evaluate and prevent radiation-related risk in space flights.

## 6. Conclusions

The microbeam has proven to be a precious and powerful tool for the investigation of non-target effects. With the improvement of microbeam techniques and the development of novel biological systems, a more comprehensive and systematic understanding of the mechanism(s) underlying RIBE and cytoplasmic irradiation will be attained in the near future. 

## Data Availability

This study did not report any data.
